# 4-(4-Nitro­benz­yl)pyridinium 3-carb­oxy-4-hy­droxy­benzene­sulfonate

**DOI:** 10.1107/S1600536813000093

**Published:** 2013-01-09

**Authors:** Graham Smith, Urs D. Wermuth

**Affiliations:** a Science and Engineering Faculty, Queensland University of Technology, GPO Box 2434, Brisbane, Queensland 4001, Australia

## Abstract

In the title salt, C_12_H_11_N_2_O_2_^+^·C_7_H_5_O_6_S^−^, the dihedral angle between the benzene and pyridine rings in the 4-(4-nitro­benz­yl)pyridinium cation is 82.7 (2)°. Within the anion there is an intramolecular hydroxy-O—H⋯O(carboxylic acid) bond. In the crystal, the cation forms a single N^+^—H⋯O_sulfonate_ hydrogen bond with the anion. These cation–anion pairs inter­act through duplex anion carb­oxy­lic acid O—H⋯O_sulfonate_ hydrogen bonds, giving a centrosymmetric cyclic association [graph set *R*_2_^2^(16)]. The crystals studied were non-merohedrally twinned.

## Related literature

For data on 4-(4-nitro­benz­yl)pyridine adduct and salt structures, see: Smith *et al.* (1997 ▶); Smith & Wermuth (2010 ▶). For examples of the structures of salts of 5-sulfosalicylic acid, see: Raj *et al.* (2003 ▶); Smith *et al.* (2004 ▶). For graph-set analysis of hydrogen bonds, see: Etter *et al.* (1990 ▶).
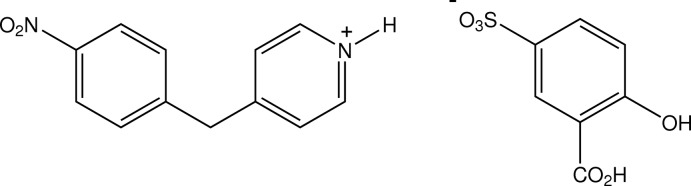


## Experimental

### 

#### Crystal data

C_12_H_11_N_2_O_2_^+^·C_7_H_5_O_6_S^−^
*M* *_r_* = 432.41
Monoclinic, 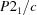

*a* = 7.4154 (7) Å
*b* = 12.8896 (10) Å
*c* = 19.649 (2) Å
β = 92.848 (9)°
*V* = 1875.8 (3) Å^3^
*Z* = 4
Mo *K*α radiation
μ = 0.23 mm^−1^
*T* = 200 K
0.25 × 0.20 × 0.15 mm

#### Data collection

Oxford Diffraction Gemini-S CCD-detector diffractometer
Absorption correction: multi-scan (*CrysAlis PRO*; Agilent, 2012 ▶) *T*_min_ = 0.916, *T*_max_ = 0.980
14534 measured reflections
3671 independent reflections
2631 reflections with *I* > 2σ(*I*)
*R*_int_ = 0.049

#### Refinement

*R*[*F* ^2^ > 2σ(*F* ^2^)] = 0.071
*wR*(*F* ^2^) = 0.173
*S* = 1.21
3671 reflections
272 parameters
H-atom parameters constrained
Δρ_max_ = 0.39 e Å^−3^
Δρ_min_ = −0.54 e Å^−3^

### 

Data collection: *CrysAlis PRO* (Agilent, 2012 ▶); cell refinement: *CrysAlis PRO*; data reduction: *CrysAlis PRO*; program(s) used to solve structure: *SIR92* (Altomare *et al.*, 1993 ▶); program(s) used to refine structure: *SHELXL97* (Sheldrick, 2008 ▶) within *WinGX* (Farrugia, 2012 ▶); molecular graphics: *PLATON* (Spek, 2009 ▶); software used to prepare material for publication: *PLATON*.

## Supplementary Material

Crystal structure: contains datablock(s) global, I. DOI: 10.1107/S1600536813000093/bh2470sup1.cif

Structure factors: contains datablock(s) I. DOI: 10.1107/S1600536813000093/bh2470Isup2.hkl

Supplementary material file. DOI: 10.1107/S1600536813000093/bh2470Isup3.cml

Additional supplementary materials:  crystallographic information; 3D view; checkCIF report

## Figures and Tables

**Table 1:** Hydrogen-bond geometry (Å, °)

*D*—H⋯*A*	*D*—H	H⋯*A*	*D*⋯*A*	*D*—H⋯*A*
N1—H1⋯O51*A*	0.86	1.88	2.732 (5)	172
O2*A*—H2*A*⋯O12*A*	0.95	1.70	2.613 (5)	159
O11*A*—H11*A*⋯O53*A* ^i^	0.94	1.65	2.583 (4)	172

Symmetry code: (i) 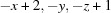.

**Fig. 1.**
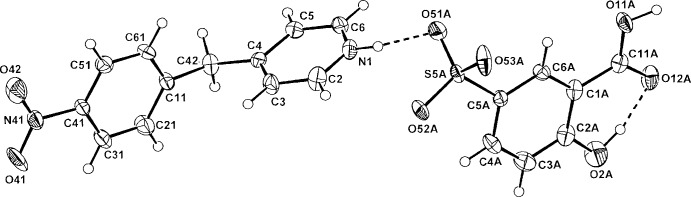
Molecular configuration and atom naming scheme for the hydrogen-bonded NBPY cation and 5-SSA monoanion species in the title salt. Hydrogen bonds are shown as dashed lines and displacement ellipsoids are drawn at the 40% probability level.

**Fig. 2.**
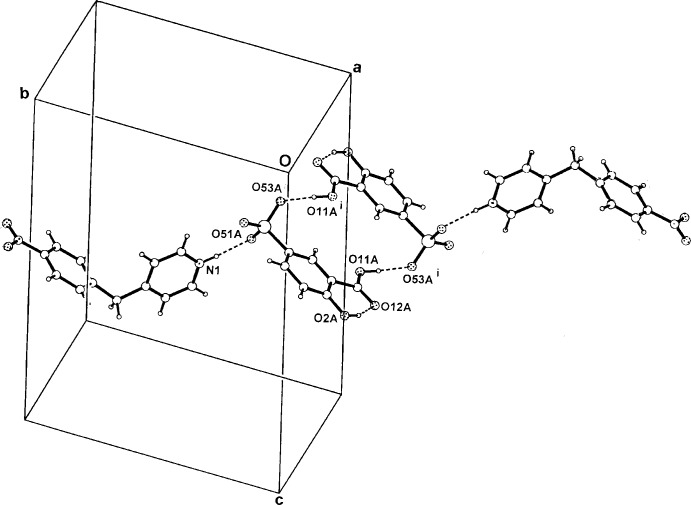
A perspective view of the crystal packing in the unit cell showing the centrosymmetric anion-associated hydrogen-bonded cation–anion pairs. For symmetry code (i), see Table 1.

## supplementary crystallographic information

### Comment

The Lewis base 4-(4-nitrobenzyl)pyridine (NBPY) is an analogue of 2-(2,4-dinitrobenzyl)pyridine (DNBPY) which is significant because of its unusual photochromic behaviour in the solid state, although NBPY does not possess such properties. The structure of NBPY is not known but both the structures of a 2:1 co-crystal adduct with 4-aminobenzoic acid (Smith *et al.*, 1997) and a 5-nitrosalicylate salt (Smith & Wermuth, 2010) have been reported. Our reaction of NBPY with 3-carboxy-4-hydroxybenzenesulfonic acid (5-sulfosalicylic acid = 5-SSA) gave the title compound, C_12_H_11_N_2_O_2_^+^ C_7_H_5_O_6_S^-^, the structure of which is reported herein. The structures of a number of 1:1 salts of 5-SSA are known (Raj *et al.*, 2003; Smith *et al.*, 2004).

With the title compound (Fig. 1), the dihedral angle between the phenyl and pyridine rings in the 4-(4-nitrobenzyl)pyridinium cation is 82.7 (2)° and this forms a single *N*^+^—*H*···O_sulfonate_ hydrogen bond with the anion. These cation–anion pairs inter-associate through duplex anion carboxylic acid *O*—*H*···O_sulfonate_ hydrogen bonds (Table 1, Fig. 2) giving a centrosymmetric cyclic motif [graph set *R*^2^_2_(16) (Etter *et al.*, 1990)]. Crystals of the compound are non-merohedrally twinned [BASF factor 0.3201 (Sheldrick, 2008); see *Refinement* section].

In the 5-SSA monoanion, the usual intramolecular phenol *O*–*H*···O_carboxyl_ hydrogen bond [2.613 (5) Å] is present, essentially maintaining coplanarity of the carboxylic acid group and the benzene ring [torsion angle C2A—C1A—C11A—O11A, -176.6 (4)°] (Raj *et al.*, 2003; Smith *et al.*, 2004).

### Experimental

The title compound was synthesized by heating together under reflux for 10 minutes, 1 mmol quantities of 4-(4-nitrobenzyl)pyridine with 5-sulfosalicylic acid in 50 ml of 50% ethanol–water. After concentration to *ca*. 30 ml, partial room temperature evaporation of the hot-filtered solution gave colourless crystals from which a block section was cleaved for the X-ray analysis.

### Refinement

Hydrogen atoms involved in hydrogen-bonding interactions were located by difference methods but their positional and isotropic displacement parameters were allowed to ride in the refinement, with *U*_iso_(H) = 1.2*U*_eq_(N) or 1.5*U*_eq_(O)]. Other H atoms were included in the refinement at calculated positions [C—H = 0.93 Å (aromatic) and 0.97 Å (aliphatic) and *U*_iso_(H) = 1.2*U*_eq_(C)], also using a riding-model approximation. The crystal was found to be non-merohedrally twinned [Twin Rot Mat [*PLATON* (Spek, 2009)]: matrix, -1 0 0, 0 - 1 0, 0.263 0 1] and the data generated were used in the final refinement (refined BASF = 0.3201).

### Crystal data

**Table:**

C_12_H_11_N_2_O_2_^+^·C_7_H_5_O_6_S^−^	*F*(000) = 896
*M**_r_* = 432.41	*D*_x_ = 1.531 Mg m^−^^3^
Monoclinic, *P*2_1_/*c*	Mo *K*α radiation, λ = 0.71073 Å
Hall symbol: -P 2ybc	Cell parameters from 3757 reflections
*a* = 7.4154 (7) Å	θ = 3.2–28.8°
*b* = 12.8896 (10) Å	µ = 0.23 mm^−^^1^
*c* = 19.649 (2) Å	*T* = 200 K
β = 92.848 (9)°	Block, colourless
*V* = 1875.8 (3) Å^3^	0.25 × 0.20 × 0.15 mm
*Z* = 4	

### Data collection

**Table:**

Oxford Diffraction Gemini-S CCD-detector diffractometer	3671 independent reflections
Radiation source: Enhance (Mo) X-ray source	2631 reflections with *I* > 2σ(*I*)
Graphite monochromator	*R*_int_ = 0.049
Detector resolution: 16.077 pixels mm^-1^	θ_max_ = 26.0°, θ_min_ = 3.2°
ω scans	*h* = −9→9
Absorption correction: multi-scan (*CrysAlis PRO*; Agilent, 2012)	*k* = −15→15
*T*_min_ = 0.916, *T*_max_ = 0.980	*l* = 0→24
14534 measured reflections	

### Refinement

**Table:**

Refinement on *F*^2^	Primary atom site location: structure-invariant direct methods
Least-squares matrix: full	Secondary atom site location: difference Fourier map
*R*[*F*^2^ > 2σ(*F*^2^)] = 0.071	Hydrogen site location: inferred from neighbouring sites
*wR*(*F*^2^) = 0.173	H-atom parameters constrained
*S* = 1.21	*w* = 1/[σ^2^(*F*_o_^2^) + (0.0464*P*)^2^ + 2.5904*P*] where *P* = (*F*_o_^2^ + 2*F*_c_^2^)/3
3671 reflections	(Δ/σ)_max_ < 0.001
272 parameters	Δρ_max_ = 0.39 e Å^−^^3^
0 restraints	Δρ_min_ = −0.54 e Å^−^^3^
0 constraints	

### Fractional atomic coordinates and isotropic or equivalent isotropic displacement parameters (Å^2^)

**Table:**

	*x*	*y*	*z*	*U*_iso_*/*U*_eq_	
O41	0.5308 (6)	1.2075 (3)	0.6714 (2)	0.0669 (15)	
O42	0.4378 (6)	1.1939 (3)	0.5666 (2)	0.0580 (16)	
N1	0.6089 (5)	0.4772 (3)	0.58094 (19)	0.0343 (14)	
N41	0.4750 (5)	1.1565 (3)	0.6224 (2)	0.0374 (14)	
C2	0.6921 (7)	0.5677 (4)	0.5906 (3)	0.0433 (19)	
C3	0.6005 (7)	0.6507 (4)	0.6162 (3)	0.0363 (16)	
C4	0.4226 (6)	0.6405 (3)	0.6324 (2)	0.0265 (14)	
C5	0.3420 (6)	0.5439 (3)	0.6213 (2)	0.0332 (16)	
C6	0.4369 (7)	0.4631 (3)	0.5959 (2)	0.0347 (16)	
C11	0.3719 (6)	0.8367 (3)	0.6512 (2)	0.0304 (16)	
C21	0.4350 (8)	0.8981 (4)	0.7051 (2)	0.0439 (19)	
C31	0.4738 (7)	1.0020 (4)	0.6955 (3)	0.0447 (19)	
C41	0.4452 (6)	1.0441 (3)	0.6322 (2)	0.0294 (16)	
C42	0.3137 (7)	0.7262 (3)	0.6619 (2)	0.0351 (16)	
C51	0.3888 (6)	0.9853 (4)	0.5774 (2)	0.0328 (16)	
C61	0.3547 (6)	0.8809 (3)	0.5871 (2)	0.0305 (14)	
S5A	0.96726 (16)	0.31180 (8)	0.53828 (6)	0.0283 (3)	
O2A	1.0084 (6)	−0.0013 (3)	0.75787 (17)	0.0573 (14)	
O11A	0.8687 (5)	−0.0882 (2)	0.55774 (16)	0.0372 (10)	
O12A	0.9140 (5)	−0.1398 (2)	0.66576 (17)	0.0427 (11)	
O51A	0.7756 (5)	0.3169 (2)	0.51826 (16)	0.0385 (11)	
O52A	1.0352 (5)	0.4070 (2)	0.56822 (18)	0.0433 (11)	
O53A	1.0709 (5)	0.2754 (2)	0.48221 (18)	0.0503 (13)	
C1A	0.9474 (6)	0.0393 (3)	0.6394 (2)	0.0317 (16)	
C2A	1.0008 (7)	0.0680 (4)	0.7061 (2)	0.0363 (16)	
C3A	1.0483 (7)	0.1698 (4)	0.7204 (2)	0.0443 (17)	
C4A	1.0432 (7)	0.2439 (4)	0.6695 (2)	0.0361 (17)	
C5A	0.9863 (6)	0.2161 (3)	0.6031 (2)	0.0250 (12)	
C6A	0.9407 (6)	0.1142 (3)	0.5884 (2)	0.0273 (14)	
C11A	0.9080 (6)	−0.0709 (3)	0.6227 (2)	0.0304 (14)	
H1	0.66760	0.42610	0.56460	0.0410*	
H2	0.81240	0.57490	0.58020	0.0520*	
H3	0.65890	0.71410	0.62250	0.0440*	
H5	0.22190	0.53430	0.63130	0.0400*	
H6	0.38210	0.39880	0.58900	0.0420*	
H21	0.45160	0.86920	0.74840	0.0520*	
H31	0.51860	1.04250	0.73170	0.0540*	
H51	0.37360	1.01480	0.53430	0.0390*	
H61	0.31950	0.83990	0.54990	0.0370*	
H421	0.31140	0.71450	0.71060	0.0420*	
H422	0.19060	0.71940	0.64330	0.0420*	
H2A	0.97480	−0.06270	0.73320	0.0860*	
H3A	1.08390	0.18860	0.76480	0.0530*	
H4A	1.07740	0.31190	0.67930	0.0430*	
H6A	0.90540	0.09560	0.54390	0.0320*	
H11A	0.88000	−0.15700	0.54300	0.0560*	

### Atomic displacement parameters (Å^2^)

**Table:**

	*U*^11^	*U*^22^	*U*^33^	*U*^12^	*U*^13^	*U*^23^
O41	0.098 (3)	0.0271 (19)	0.074 (3)	−0.016 (2)	−0.012 (3)	−0.020 (2)
O42	0.082 (3)	0.033 (2)	0.059 (3)	−0.008 (2)	0.004 (2)	0.0134 (19)
N1	0.040 (3)	0.027 (2)	0.036 (2)	0.0075 (19)	0.0044 (18)	0.0012 (18)
N41	0.034 (2)	0.024 (2)	0.054 (3)	−0.0013 (18)	−0.001 (2)	−0.006 (2)
C2	0.032 (3)	0.034 (3)	0.065 (4)	0.000 (2)	0.013 (3)	0.008 (3)
C3	0.039 (3)	0.020 (2)	0.050 (3)	−0.004 (2)	0.004 (2)	0.003 (2)
C4	0.033 (3)	0.021 (2)	0.025 (2)	−0.002 (2)	−0.0044 (19)	0.0075 (18)
C5	0.030 (3)	0.031 (2)	0.038 (3)	−0.002 (2)	−0.003 (2)	−0.003 (2)
C6	0.043 (3)	0.023 (2)	0.037 (3)	−0.003 (2)	−0.008 (2)	0.001 (2)
C11	0.033 (3)	0.023 (2)	0.035 (3)	0.002 (2)	0.001 (2)	−0.007 (2)
C21	0.069 (4)	0.035 (3)	0.027 (3)	−0.002 (3)	−0.004 (2)	0.000 (2)
C31	0.066 (4)	0.028 (3)	0.039 (3)	0.000 (3)	−0.009 (3)	−0.011 (2)
C41	0.029 (3)	0.023 (2)	0.036 (3)	0.001 (2)	−0.001 (2)	−0.008 (2)
C42	0.040 (3)	0.026 (2)	0.040 (3)	0.003 (2)	0.008 (2)	0.001 (2)
C51	0.036 (3)	0.031 (2)	0.031 (3)	−0.002 (2)	−0.001 (2)	0.001 (2)
C61	0.035 (3)	0.030 (2)	0.026 (2)	−0.006 (2)	−0.002 (2)	−0.007 (2)
S5A	0.0326 (6)	0.0160 (5)	0.0364 (6)	−0.0023 (5)	0.0040 (5)	−0.0030 (5)
O2A	0.093 (3)	0.051 (2)	0.0285 (19)	0.004 (2)	0.0092 (19)	0.0112 (18)
O11A	0.059 (2)	0.0190 (15)	0.0336 (18)	−0.0051 (16)	0.0011 (16)	0.0005 (14)
O12A	0.059 (2)	0.0300 (18)	0.040 (2)	0.0037 (18)	0.0116 (17)	0.0138 (16)
O51A	0.046 (2)	0.0291 (17)	0.0398 (19)	−0.0026 (17)	−0.0037 (15)	−0.0011 (15)
O52A	0.049 (2)	0.0192 (16)	0.061 (2)	−0.0087 (16)	−0.0056 (18)	−0.0087 (16)
O53A	0.079 (3)	0.0230 (16)	0.052 (2)	0.0096 (18)	0.036 (2)	0.0085 (16)
C1A	0.034 (3)	0.028 (2)	0.034 (3)	0.003 (2)	0.011 (2)	0.005 (2)
C2A	0.047 (3)	0.037 (3)	0.026 (2)	0.010 (2)	0.013 (2)	0.004 (2)
C3A	0.057 (3)	0.049 (3)	0.027 (3)	0.002 (3)	0.002 (2)	−0.011 (2)
C4A	0.039 (3)	0.031 (3)	0.039 (3)	−0.004 (2)	0.009 (2)	−0.013 (2)
C5A	0.022 (2)	0.021 (2)	0.032 (2)	−0.0002 (18)	0.0023 (19)	−0.0011 (18)
C6A	0.031 (3)	0.028 (2)	0.023 (2)	−0.002 (2)	0.0027 (19)	−0.0013 (19)
C11A	0.026 (2)	0.031 (2)	0.035 (3)	0.002 (2)	0.009 (2)	0.005 (2)

### Geometric parameters (Å, º)

**Table:**

S5A—C5A	1.774 (4)	C31—C41	1.364 (7)
S5A—O53A	1.452 (4)	C41—C51	1.365 (6)
S5A—O51A	1.457 (4)	C51—C61	1.384 (6)
S5A—O52A	1.441 (3)	C2—H2	0.9300
O41—N41	1.221 (6)	C3—H3	0.9300
O42—N41	1.217 (6)	C5—H5	0.9300
O2A—C2A	1.353 (6)	C6—H6	0.9300
O11A—C11A	1.314 (5)	C21—H21	0.9300
O12A—C11A	1.226 (5)	C31—H31	0.9300
O2A—H2A	0.9500	C42—H422	0.9700
O11A—H11A	0.9400	C42—H421	0.9700
N1—C6	1.336 (6)	C51—H51	0.9300
N1—C2	1.329 (6)	C61—H61	0.9300
N41—C41	1.480 (5)	C1A—C11A	1.484 (6)
N1—H1	0.8600	C1A—C2A	1.400 (6)
C2—C3	1.376 (7)	C1A—C6A	1.391 (6)
C3—C4	1.379 (7)	C2A—C3A	1.384 (7)
C4—C5	1.394 (6)	C3A—C4A	1.382 (6)
C4—C42	1.502 (6)	C4A—C5A	1.398 (6)
C5—C6	1.365 (6)	C5A—C6A	1.383 (6)
C11—C21	1.385 (6)	C3A—H3A	0.9300
C11—C42	1.506 (6)	C4A—H4A	0.9300
C11—C61	1.383 (6)	C6A—H6A	0.9300
C21—C31	1.385 (7)		
			
O51A—S5A—O53A	110.8 (2)	C6—C5—H5	119.00
O51A—S5A—C5A	105.49 (19)	N1—C6—H6	120.00
O52A—S5A—O53A	113.4 (2)	C5—C6—H6	120.00
O52A—S5A—C5A	106.6 (2)	C11—C21—H21	120.00
O53A—S5A—C5A	107.09 (19)	C31—C21—H21	119.00
O51A—S5A—O52A	112.85 (19)	C21—C31—H31	120.00
C2A—O2A—H2A	100.00	C41—C31—H31	120.00
C11A—O11A—H11A	116.00	C4—C42—H422	108.00
C2—N1—C6	122.0 (4)	C11—C42—H421	108.00
O41—N41—O42	123.4 (4)	C11—C42—H422	108.00
O42—N41—C41	118.5 (4)	H421—C42—H422	107.00
O41—N41—C41	118.1 (4)	C4—C42—H421	108.00
C6—N1—H1	119.00	C41—C51—H51	121.00
C2—N1—H1	119.00	C61—C51—H51	121.00
N1—C2—C3	120.1 (5)	C11—C61—H61	119.00
C2—C3—C4	120.4 (5)	C51—C61—H61	119.00
C5—C4—C42	118.9 (4)	C6A—C1A—C11A	120.4 (4)
C3—C4—C5	117.2 (4)	C2A—C1A—C11A	120.2 (4)
C3—C4—C42	123.9 (4)	C2A—C1A—C6A	119.3 (4)
C4—C5—C6	120.9 (4)	O2A—C2A—C3A	118.2 (4)
N1—C6—C5	119.5 (4)	O2A—C2A—C1A	121.9 (4)
C21—C11—C42	121.5 (4)	C1A—C2A—C3A	119.9 (4)
C21—C11—C61	118.3 (4)	C2A—C3A—C4A	120.7 (4)
C42—C11—C61	120.2 (4)	C3A—C4A—C5A	119.6 (4)
C11—C21—C31	120.9 (4)	C4A—C5A—C6A	119.8 (4)
C21—C31—C41	119.0 (5)	S5A—C5A—C4A	120.1 (3)
N41—C41—C31	119.4 (4)	S5A—C5A—C6A	120.0 (3)
N41—C41—C51	118.9 (4)	C1A—C6A—C5A	120.6 (4)
C31—C41—C51	121.7 (4)	O12A—C11A—C1A	122.8 (4)
C4—C42—C11	118.6 (4)	O11A—C11A—O12A	123.1 (4)
C41—C51—C61	118.8 (4)	O11A—C11A—C1A	114.1 (3)
C11—C61—C51	121.1 (4)	C2A—C3A—H3A	120.00
N1—C2—H2	120.00	C4A—C3A—H3A	120.00
C3—C2—H2	120.00	C3A—C4A—H4A	120.00
C4—C3—H3	120.00	C5A—C4A—H4A	120.00
C2—C3—H3	120.00	C1A—C6A—H6A	120.00
C4—C5—H5	120.00	C5A—C6A—H6A	120.00
			
O53A—S5A—C5A—C6A	−54.6 (4)	C42—C11—C61—C51	172.9 (4)
O52A—S5A—C5A—C4A	5.3 (4)	C11—C21—C31—C41	1.4 (8)
O53A—S5A—C5A—C4A	126.9 (4)	C21—C31—C41—N41	176.1 (5)
O51A—S5A—C5A—C4A	−114.9 (4)	C21—C31—C41—C51	−3.6 (8)
O52A—S5A—C5A—C6A	−176.3 (4)	C31—C41—C51—C61	2.0 (7)
O51A—S5A—C5A—C6A	63.5 (4)	N41—C41—C51—C61	−177.7 (4)
C6—N1—C2—C3	−0.6 (8)	C41—C51—C61—C11	1.9 (7)
C2—N1—C6—C5	0.6 (7)	C2A—C1A—C11A—O11A	−176.6 (4)
O41—N41—C41—C31	2.5 (6)	C2A—C1A—C11A—O12A	2.6 (7)
O42—N41—C41—C51	4.3 (6)	C6A—C1A—C11A—O11A	0.1 (6)
O41—N41—C41—C51	−177.8 (4)	C6A—C1A—C11A—O12A	179.2 (4)
O42—N41—C41—C31	−175.4 (5)	C11A—C1A—C2A—O2A	−3.8 (7)
N1—C2—C3—C4	0.4 (8)	C11A—C1A—C2A—C3A	175.9 (4)
C2—C3—C4—C42	178.7 (5)	C2A—C1A—C6A—C5A	0.0 (7)
C2—C3—C4—C5	−0.4 (7)	C11A—C1A—C6A—C5A	−176.7 (4)
C3—C4—C5—C6	0.5 (6)	C6A—C1A—C2A—O2A	179.4 (5)
C42—C4—C5—C6	−178.6 (4)	C6A—C1A—C2A—C3A	−0.8 (7)
C3—C4—C42—C11	22.4 (6)	O2A—C2A—C3A—C4A	180.0 (5)
C5—C4—C42—C11	−158.6 (4)	C1A—C2A—C3A—C4A	0.2 (8)
C4—C5—C6—N1	−0.6 (6)	C2A—C3A—C4A—C5A	1.2 (8)
C61—C11—C42—C4	69.8 (6)	C3A—C4A—C5A—C6A	−1.9 (7)
C21—C11—C61—C51	−3.9 (7)	C3A—C4A—C5A—S5A	176.5 (4)
C21—C11—C42—C4	−113.5 (5)	S5A—C5A—C6A—C1A	−177.2 (3)
C42—C11—C21—C31	−174.6 (5)	C4A—C5A—C6A—C1A	1.3 (7)
C61—C11—C21—C31	2.3 (8)		

### Hydrogen-bond geometry (Å, º)

**Table:**

*D*—H···*A*	*D*—H	H···*A*	*D*···*A*	*D*—H···*A*
N1—H1···O51*A*	0.86	1.88	2.732 (5)	172
O2*A*—H2*A*···O12*A*	0.95	1.70	2.613 (5)	159
O11*A*—H11*A*···O53*A*^i^	0.94	1.65	2.583 (4)	172
C2—H2···O53*A*^ii^	0.93	2.47	3.078 (6)	123
C3*A*—H3*A*···O12*A*^iii^	0.93	2.60	3.323 (6)	135
C4*A*—H4*A*···O52*A*	0.93	2.51	2.893 (6)	105
C5—H5···O52*A*^iv^	0.93	2.44	3.024 (5)	120
C6—H6···O52*A*^iv^	0.93	2.59	3.087 (6)	114
C6*A*—H6*A*···O11*A*	0.93	2.40	2.724 (5)	100
C61—H61···O51*A*^v^	0.93	2.51	3.394 (5)	160
C42—H421···O41^vi^	0.97	2.55	3.427 (6)	151

Symmetry codes: (i) −*x*+2, −*y*, −*z*+1; (ii) −*x*+2, −*y*+1, −*z*+1; (iii) −*x*+2, *y*+1/2, −*z*+3/2; (iv) *x*−1, *y*, *z*; (v) −*x*+1, −*y*+1, −*z*+1; (vi) −*x*+1, *y*−1/2, −*z*+3/2.

## References

[bb1] Agilent (2012). *CrysAlis PRO* Agilent Technologies Ltd, Yarnton, England.

[bb2] Altomare, A., Cascarano, G., Giacovazzo, C. & Guagliardi, A. (1993). *J. Appl. Cryst.* **26**, 343–350.

[bb3] Etter, M. C., MacDonald, J. C. & Bernstein, J. (1990). *Acta Cryst.* B**46**, 256–262. 10.1107/s0108768189012929 2344397

[bb4] Farrugia, L. J. (2012). *J. Appl. Cryst.* **45**, 849–854.

[bb5] Raj, S. B., Sethuraman, V., Francis, S., Hemamalini, M., Muthiah, P. T., Bocelli, G., Cantoni, A., Rychlewska, U. & Warzajtis, B. (2003). *CrysEngComm*, **5**, 70–76.

[bb6] Sheldrick, G. M. (2008). *Acta Cryst.* A**64**, 112–122. 10.1107/S0108767307043930 18156677

[bb7] Smith, G., Lynch, D. E., Byriel, K. A. & Kennard, C. H. L. (1997). *J. Chem. Crystallogr.* **27**, 307–317.

[bb8] Smith, G. & Wermuth, U. D. (2010). *Acta Cryst.* E**66**, o1173. 10.1107/S1600536810014698 PMC2979186 21579214

[bb9] Smith, G., Wermuth, U. D. & White, J. M. (2004). *Acta Cryst.* C**60**, o575–o581. 10.1107/S010827010401457X 15295192

[bb10] Spek, A. L. (2009). *Acta Cryst.* D**65**, 148–155. 10.1107/S090744490804362X PMC2631630 19171970

